# Mesoscopic Perspective into the High-Temperature Triaxial Dilation of Asphalt Mixtures via PFC–FLAC Coupled Simulation

**DOI:** 10.3390/ma18081722

**Published:** 2025-04-09

**Authors:** Bin Xiao, Wei Cao, Liang Zhou

**Affiliations:** 1 School of Civil Engineering, Central South University, Changsha 410078, China; 224812251@csu.edu.cn; 2 Hunan Junjia Pavement Material Co., Ltd., Changsha 410000, China; 18173259575@163.com

**Keywords:** triaxial strength test, shear dilation, PFC–FLAC coupled simulation, aggregate movement, contact failures, anisotropy of contact forces

## Abstract

The high-temperature rutting performance of asphalt mixtures is strongly dependent on the aggregate skeleton and particle movement under loading. Such mechanisms were addressed in the present study by a combined experimental and simulation approach based on the triaxial strength test. A single type of asphalt with two different aggregate gradations (dense and gap) was incorporated to highlight the role of gradation in resisting shear dilation. The simulation was carried out by coupling the discrete and finite element methods considering the realistic three-dimensional aggregate shapes and gradations as well as the flexible boundary prescribed by latex membranes as routinely employed in triaxial testing. In order to represent contact failure-induced cracks within the virtual specimens, the linear parallel bond model was mixed with the Burgers or linear model through random distribution at contacts involving the mortar units. Model verification was achieved by comparing the resulting stress–strain data against those from the laboratory. The calibrated model provided a platform for systematic investigation from the perspectives of particle movement, crack development and distribution, and interparticle contacts. The results showed that the gap-graded mixture yielded lower triaxial strengths and yet softened at a lower rate and exhibited smaller volumetric expansion in the post-peak region. A faster loss of internal cohesion was inferred in the dense-graded mixture based on the higher accumulation rate of cracks that were concentrated at the middle height towards the perimeter of the virtual specimen. Contact analysis indicated that aggregate skeleton was more influential in the strength and stability of gap-graded mixtures.

## 1. Introduction

With the increasing traffic volume and global warming, the rutting distress in asphalt pavement is becoming increasingly serious in general [1]. Rutting is mainly caused by the insufficient shear resistance of asphalt mixtures at high temperatures, resulting in depression at the wheel path with humps at both sides that are attributed to the particle flow under shearing [2,3]. To deeply understand the rutting formation mechanism as well as the shear behavior, different experimental methods have been adopted, including the simple punching shear test (SPST) and the repeated loading test with or without lateral confinement [4,5,6,7]. Among them, the triaxial test provides a closer approximation to the actual three-dimensional stress state in pavement structures, while repeated loading mimics the field trafficking pattern more realistically than the monotonic test [8,9]. Through the triaxial strength test, Gu et al. [10] identified the internal cohesion *c* and friction angle *φ*, and the results indicated that a larger nominal maximum aggregate size (NMAS) yielded a higher friction angle. The authors further employed the CoreLok method to quantify the volumetric changes of the test specimens and noted that the degree of dilation was reduced at higher confinement. Based on the radial deformation measured by specialized displacement transducers, the dilation behavior was found to be primarily influenced by the kinematic constraints imposed by the aggregate skeleton [11].

In addition to the traditional laboratory efforts, numerical simulation has become an effective approach to providing smaller-scale insights into material behaviors, and in this respect, the finite and discrete element methods have stood out as the leading tools. Generally speaking, the former is more focused on the structural mechanical responses [12,13], whereas the latter appears to be more advantageous in dealing with large deformations and cracking of highly heterogeneous composite materials [14,15]. The aforementioned experimental characterization methods have mostly been implemented in discrete element simulations [16,17,18,19,20,21]. These studies have mainly been focused on evaluating the impacts of different factors affecting the shear strength and other performance indicators, with less attention on the evolution characteristics of deformation along the loading history. For instance, Collop et al. [22] investigated the dependence of the dilation behavior of asphalt mixtures on the contact parameters of the discrete element model (DEM) and reported that increasing the ratio of compressive to tensile stiffness over time led to increased axial deformation. Zhang et al. [11] experimentally identified the significant roles of the asphalt binder softening point and aggregate structure in the dilation behavior, in agreement with their DEM observations, but an in-depth investigation into the aggregate particle movement (which is deemed highly pertinent to dilation) was not provided. Fan et al. [23] evaluated the impacts of loading rate, confining pressure, pore characteristics, and aggregate properties. They reported that faster loading and higher pressure would reduce dilation, whereas a larger porosity led to greater dilation. Additionally, a higher aggregate content and rougher particle surfaces would contribute to a larger dilation angle.

The triaxial strength test is one of the most widely employed approaches to characterize the high-temperature strength and deformation behavior of asphalt mixtures, and yet there has been a significant inconsistency between the test and DEM simulations in the existing studies. In simulations, the confining pressure has usually been applied through rigid walls to the specimen, imposing a uniform lateral deformation as opposed to the flexible boundary condition in reality [23]. In the actual testing, the cylindrical specimen would tend to bulge at the middle height, whereas towards the two ends the lateral expansion is gradually suppressed. This inconsistency would result in the incompetence of DEM to reproduce the shear failure in the triaxial test. As a remedy, a particle wall was devised to replace the rigid ones [24], but with the increased number of particles comes added model complexity and computational expense. In recent years, the discrete element–finite element coupling strategy has emerged as a new attempt to simulate asphalt mixtures (as discrete elements) under loading by moving wheels or vibratory compactors (as finite elements) [25,26]. This approach has also demonstrated its effectiveness in modeling the deformation and failure of soil–rock mixtures under confinement applied to the flexible boundaries [27,28].

Aimed at insight into the dilation behavior, which is of critical significance to the high-temperature performance of asphalt mixtures, the present study simulated a triaxial strength test considering both the composite nature of the materials and the flexible lateral boundary by taking advantage of the coupled discrete and finite element methodology. The dilation behavior was characterized from the mesoscopic perspectives of aggregate structure, particle movement, and interparticle contacts, with the additional incorporation of contact failures that allowed for describing the post-peak softening phase.

## 2. Materials and Experimental Method

### 2.1. Materials

**Figure 1:**
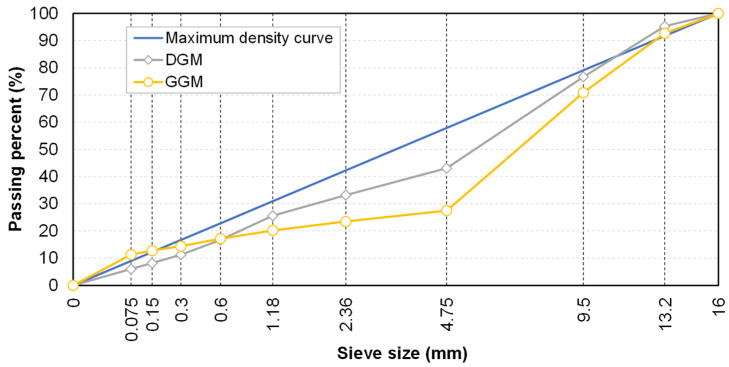
Gradation curves of the DGM and GGM.

Aggregate gradation was identified as the primary factor influencing the dilation behavior of asphalt mixtures. To minimize complexity caused by other influencing factors, the same types of asphalt binder and aggregate were used. The asphalt binder selected was a #70 penetration-grade petroleum asphalt. Limestone was used as fine aggregate and mineral powder for the 0–5 mm sieve size range, while basalt was used as coarse aggregate (above 5 mm), all materials were sourced from a local manufacturer in Changsha. The material properties were tested according to the standard specifications [29,30], with the results listed in Table 1 and Table 2, respectively. In order to evaluate the impact of the aggregate skeleton on dilation behavior, two gradation types with the same nominal maximum aggregate size (NMAS) of 13 mm were examined: a conventional dense-graded mixture (DGM) and a gap-graded mixture (GGM), as illustrated in Figure 1.

Dense gradation (e.g., Superpave mixtures) has been widely employed for its adequate durability and resistance to deformation, while gap gradation (e.g., stone matrix asphalt) is known for its excellent stability and resistance to rutting under heavy traffic loads. Both mixture types are commonly used in surface layers where rutting is most prevalent due to direct exposure to traffic and the environment. It is of interest to understand how these two different gradations would affect the high-temperature performance from a mesoscopic perspective. The optimum asphalt–aggregate ratios were determined according to the Marshall mix design method as 4.8% and 5.8% for the DGM and the GGM, respectively, with target air voids of 4%.

### 2.2. Triaxial Testing

**Figure 2:**
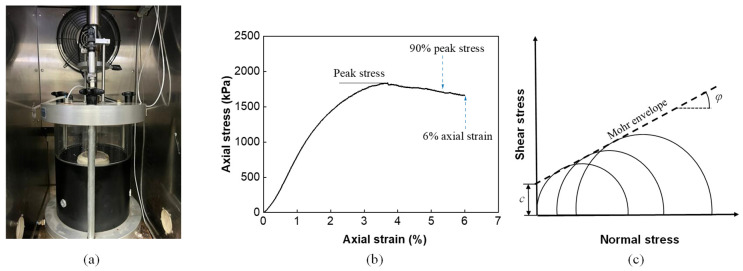
Triaxial strength test: (**a**) specimen setup in the triaxial cell, (**b**) a typical stress–strain curve and the test stopping criterion, (**c**) determination of the Mohr–Coulomb failure envelope.

The experimental procedure adhered to the standard triaxial strength test method outlined in JTG T0718/E20-2011 [29]. A cylindrical test specimen with the dimensions of 100 mm diameter and 150 mm height was set up in a triaxial cell placed inside an environmental chamber for 5 h to stabilize the temperature at 60 °C. A closed-loop servo-hydraulic universal testing machine (UTM) was employed for the axial loading at a rate of 7.5 mm/min, as shown in Figure 2a. Three different levels of confining pressure at 0, 138, and 276 kPa were applied to the latex membrane-enclosed specimen by means of compressed air. For each mixture type (DGM and GGM), two replicate samples were prepared and tested under the identical conditions, and consistent results were yielded. Testing was terminated when the load dropped to 90% of the peak load or when the axial strain exceeded 6%, as shown in Figure 2b. It is noted that the axial strain was estimated using the actuator displacement, given the relatively large deformations that could not be accommodated by typical on-specimen displacement transducers. The axial peak stresses under different confining pressures were analyzed to determine the internal cohesion and friction angle based on the Mohr–Coulomb strength theory, as depicted in Figure 2c.

## 3. Discrete and Finite Element Modeling

### 3.1. Virtual Specimen

**Figure 3:**
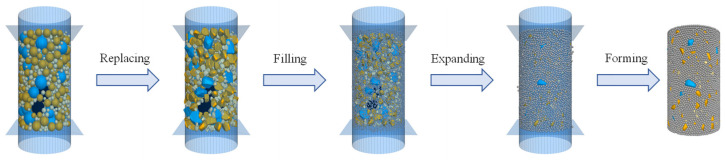
The process of virtual specimen creation.

The modeling and simulation in this study were performed using the three-dimensional particle flow code (PFC3D) [31]. The virtual specimen of the asphalt mixture comprises three components: coarse aggregate, asphalt mortar, and pores. Among these constituents, the reconstruction of coarse aggregate particles (larger than 2.36 mm) in terms of their realistic 3D shapes is of critical significance for reproducing the mechanical responses in numerical simulation [32]. Additionally, the shape characteristics of aggregates significantly affect the specimen formation process and compaction performance. For instance, aggregates with higher roundness and regularity tend to rearrange more efficiently during compaction, enhancing the initial density and compaction efficiency of the mixture [33]. Historically, CT scanning has been widely used to characterize the internal irregular aggregates of specimens [34]. An alternative effective approach for this purpose is to use a stereo lithography (STL) file (obtained via scanning equipment such as a 3D blue-ray scanner), which stores the actual shape data of particles [35]. The same approach was adopted herein, but for improved computational efficiency the STL file was properly simplified. Rigid block units were utilized to represent aggregate particles in the discrete element model. A rigid block is defined as a single piece unit, while a clump comprises multiple pebbles (i.e., individual piece units). Given that contacts are exclusively established between piece units, the adoption of rigid blocks inherently reduces the number of contacts compared to clumps. This reduction in contact count substantially enhances computational efficiency by minimizing contact retrieval time and optimizing resource utilization during simulation [31]. Asphalt mortar is composed of asphalt binder, mineral filler, and fine aggregate particles (below 2.36 mm). This component can be deemed as the continuous phase and is commonly represented by uniform ball units in DEM simulation [36]. Based on the previous studies, a radius of 1 mm was selected for these ball units [37]. The steps for creating the virtual specimen are depicted in Figure 3 and described below.

Given the computational expense, the size of the virtual specimen was reduced to 60 mm in diameter and 90 mm in height, maintaining the same dimension ratio as the actual test sample. It has been reported that smaller sizes would tend to exhibit higher compressive strengths [38,39], and incorporating size effects enables more accurate prediction of the mesoscopic parameters of the contact model [40]. The reduced specimen geometry was essentially a balance or compromise that aligns with the study’s focus, allowing for a practical mesoscopic investigation of aggregate evolution and failure mechanisms within the specimen. The number of balls required for each sieve size of aggregate was determined according to Equations (1)–(3), and the results are given in Table 3 for the DGM as an example:

(1) $$a=\frac{m_{1}}{m_{2}+m_{3}}$$

(2) $$V_{i}=\frac{P_{i+1}-P_{i}}{100}\times \left(1-VV\right)/\left(1+a\rho_{a}/\rho_{0}\right)$$

(3) $$N_{i}=V_{i}/\left(4\pi r_{i}^{3}/3\right)$$

where *a* is the asphalt–aggregate ratio; *m*_1_, *m*_2_, and *m*_3_ are the masses of asphalt binder, coarse aggregate, and fine aggregate, respectively; *V_i_* and *P_i_* are the volume fraction and the passing percentage of the *i*-th grade of aggregate, respectively; *VV* is air void ratio; *ρ_a_* and *ρ*_0_ are the densities of coarse aggregate and asphalt, respectively; and *N_i_* and *r_i_* are the number and average radius of balls for the *i*-th grade of aggregate, respectively.

The virtual specimen was generated following a procedure of graduated expansion. Initially, balls of various sizes corresponding to the aggregate gradation were created within the cylindrical region, and the model was equilibrated to eliminate ball overlaps and minimize contact forces. The STL file was then imported into PFC3D to generate the aggregate templates for each sieve size, which iterated over the balls for replacement using rigid block units with volumes equal to the balls. Subsequently, mortar ball units were employed to fill the remaining space in the cylinder to achieve the target air void content. In the final step, the sizes of mortar balls and rigid block units (for aggregate) were reduced to eliminate overlapping and then gradually recovered during equilibration.

### 3.2. Boundary Condition and the Coupled Model

**Figure 4:**
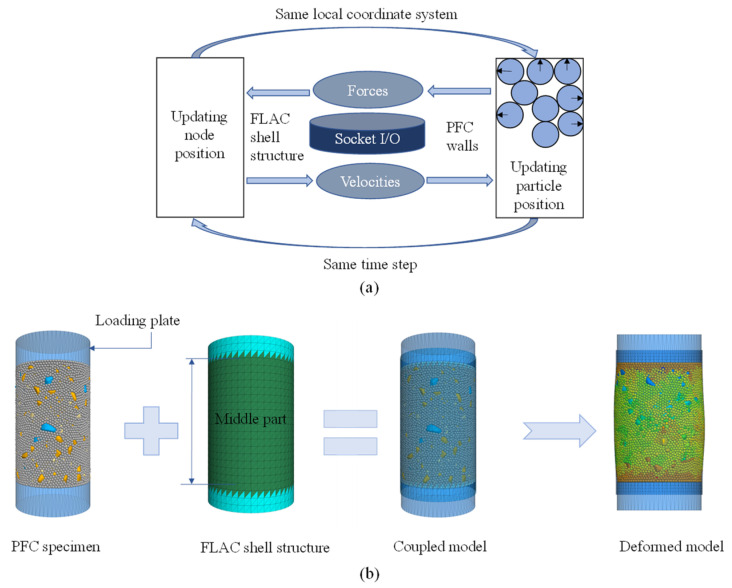
The PFC–FLAC coupled model: (**a**) the coupling principle for treating the flexible boundary, and (**b**) generation of the coupled model for simulation.

The lateral flexible boundary condition (provided by the latex membrane) of the triaxial specimen was treated via finite elements by means of Itasca’s Fast Lagrangian Analysis of Continua (FLAC) program [31]. Its coupling with PFC (for describing the enclosed heterogeneous asphalt mixture) was accomplished via the Socket O/I interface for synchronous data transmission and exchange, as outlined in Figure 4a.

Specifically, the flexible boundary was represented by a continuum of shell units for coupled computation. The continuous and discrete regions share the same coordinate system and time step for the purpose of data exchange. The FLAC boundary updates the velocities of the grid nodes at each time step and transmits them to the corresponding wall endpoints in the PFC model via the socket interface, which ensures consistent velocities between the nodes and endpoints and hence the continuous displacement field. The motion of wall endpoints causes the DEM particles to translate and rotate, generating new contact arrangements. The contact forces and bending moments are computed intrinsically by the adopted contact model and then transmitted to the grid nodes. Therefore, the contact force of the PFC model is maintained equivalent to the pressure applied to the shell continuum, ensuring stress continuity at the boundary.

The coupled model consists of the following three components: (a) top and bottom cylindrical loading plates with a diameter of 60 mm and height of 30 mm, (b) a shell structure representing the flexible membrane with a diameter of 60 mm and height of 108 mm, and (c) the virtual specimen between the two loading plates inside the shell, designated as the “middle part,” with a height of 90 mm, as seen in Figure 4b.

### 3.3. Contact Model and Parameters

**Figure 5:**
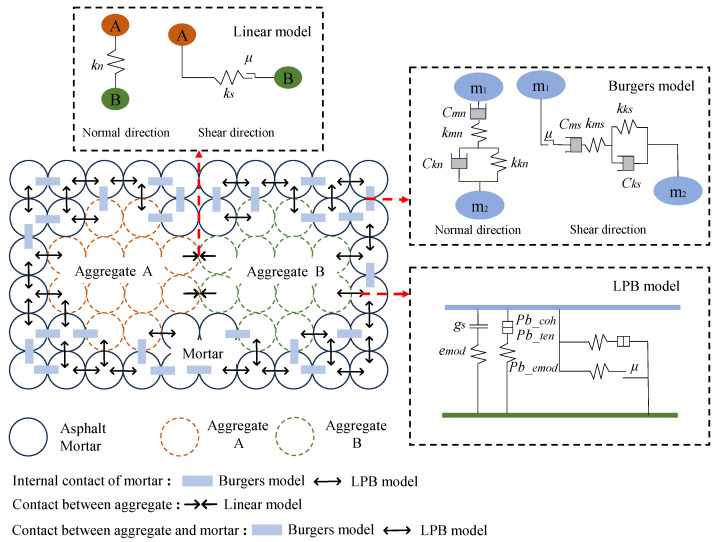
Assignment of the linear, LPB, and Burgers contact models [43].

Three contact types were considered within the virtual specimen: aggregate–aggregate, mortar–aggregate, and mortar–mortar. The aggregate–aggregate elastic contacts were described by the linear contact model. To represent contacts involving the binding phase of asphalt mortar, the Burgers model and the linear parallel bond (LPB) model have been utilized in previous studies [41]. The Burgers model effectively captures the time and temperature dependence of material responses; however, the inherent PFC model cannot describe the bonding behavior of mortar elements. On the other hand, the LPB model can be used to simulate the tensile contact forces exerted by asphalt mortar but is incapable of capturing nonlinear behaviors, especially at high temperatures [42]. For a realistic representation of the overall responses, these two models were mixed and assigned to the contacts of mortar–aggregate and mortar–mortar in a random fashion but with a specified proportion [43]. Figure 5 illustrates the assignment of the three types of contact models considered in the simulation.

The main parameters of the linear model are normal stiffness, tangential stiffness, and Poisson’s ratio, as given in Equations (4) and (5):

(4) $$k_{n}=AE^{*}/L$$

(5) $$k^{*}=\frac{k_{n}}{k_{s}}$$

where *k_n_* and *k_s_* are the normal and tangential stiffnesses of the contacts, respectively; k^*^ is the Poisson’s ratio (taken as 0.25); *A* is the area of the contact plane, given as *A* = *pr^2^*, where *r* = min (*R*_1_, *R*_2_), with *R*_1_ and *R*_2_ representing the radii of the two elements in contact; E^*^ is the effective modulus of the aggregate particle (taken as 55 GPa); and *L* is the distance between the two elements, given as *L* = *R*_1_ + *R*_2_.

Asphalt mixtures exhibit significant viscoelastic and plastic behaviors at high temperatures, which play a critical role in determining their performance under thermal and mechanical loading conditions. The implementation of the Burgers model in DEM has been shown to effectively predict the dynamic modulus and phase angle across a range of frequencies and temperatures. The Maxwell unit inherent in the Burgers model serves as an approximate description of the irreversible responses. Such capabilities provide the basis for capturing the material’s response to external loading at different temperature conditions. At increasing temperatures, the viscosity parameters of the model would decrease, resulting in enhanced fluidity and reduced viscous behaviors. The characteristic times associated with the Maxwell and Kelvin units in the Burgers model are also decreased accordingly. The overall responses of the asphalt mixture would then be controlled by the aggregate skeleton with lower time dependence. Additionally, earlier studies have reported that the Maxwell unit had a more significant influence on the predicted moduli than the Kelvin unit, underscoring the importance of prioritizing the Maxwell unit when calibrating the modulus behaviors [44].

The mesoscopic parameters of the Burgers model can be determined using the elastic beam equivalence conversion equations that have been detailed in previous studies [45]. Considering the similarities in the mixture composition and loading condition, the model parameters available in the earlier work by Xue et al. [17] were taken as the initial guesses in calibrating the Burgers model here. The LPB model parameters were determined through a trial-and-error process in conjunction with the Burgers model by fitting the experimental stress–strain curves. The mesoscopic parameters obtained for these two models are listed in Table 4 and Table 5, and the physical significances of the model parameters are illustrated in Figure 5.

### 3.4. Loading Conditions

Subsequent to its generation, the virtual specimen was symmetrically loaded at both the top and the bottom plates at a displacement rate of 0.0625 m/s. The confining pressure of 138 kPa was applied to the coupled model through the flexible shell elements. During the loading process, the stress–strain curves, aggregate movement, and generation/failure of contacts were all recorded for subsequent mesoscopic analysis.

## 4. Results and Discussion

### 4.1. Test Results and Model Verification

The triaxial compressive strength test data for the two types of mixtures at different confining conditions were processed to yield the strengths as well as the internal cohesion *c* and friction angle *j* in accordance with Figure 2c, with the results listed in Table 6. The experimental results exhibited moderate variability, with the relative standard deviation (RSD) ranging from 2.95% to 12.27%. Moreover, despite the use of duplicate specimens inherently limiting statistical precision, the numerical simulations demonstrated strong correspondence with the experimental data (with mean absolute errors < 2%). For both mixtures, an increase in the confining pressure resulted in consistently strengthened materials, as expected. Additionally, this strengthening effect was attenuated with the increasing pressure, as the incremental effect of confinement in enhancing the aggregation skeleton progressed towards saturation [46]. Between the two mixtures, the DGM consistently exhibited higher strengths than the GGM across all pressure levels. This superior performance of the DGM is also reflected in its higher internal cohesion. Recall that the same asphalt binder was employed in both mixtures for the purpose of highlighting the role of aggregate gradation. The lower strength and internal cohesion of the GGM were attributed to its higher binder content without being compensated for by an enhanced viscosity (e.g., through polymer modification). Despite these differences, the two mixtures yielded comparable friction angles, as seen in Table 6.

**Figure 6:**
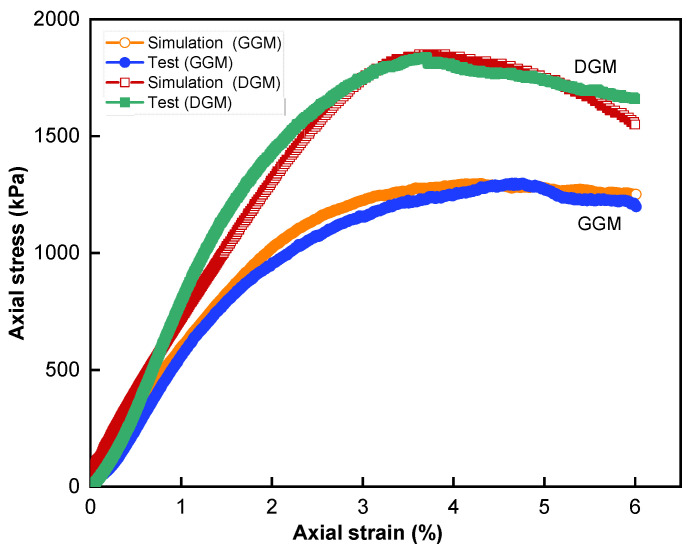
Comparison of triaxial test and simulation.

Figure 6 illustrates the comparison of stress–strain curves between the triaxial test and simulation for the confining pressure of 138 kPa. A satisfactory agreement between the experimental and simulated curves for both mixtures was observed, validating the finite- and discrete-element coupling method as well as the mixed contact model strategy. The overall deformation behaviors of the two mixtures were similar, with both exhibiting an initial densification phase before reaching peak stress, followed by a softening phase [47]. During the initial phase, the materials underwent viscoelastic–plastic deformation under increasing load, resulting in densification of the aggregate skeleton and enhanced interparticle contacts to better resist external forces. Upon reaching the peak stresses, the aggregate structures were considerably disturbed, and it was speculated that significant contact failures and internal damage were taking place. The materials then entered the post-peak softening phase, featuring increasingly pronounced shear dilatancy and eventually failure due to shear cracks.

Compared to the DGM, the use of the gap gradation exhibited a lower strength, as seen in Table 6. Yet, it should be noted that the peak stress of the GGM was reached at a considerably larger strain level in the test, suggesting a more ductile behavior during failure. This higher ductility could be attributed to the higher asphalt content. Another notable observation relates to the post-peak phase, where the stress in the GGM decreased at a much slower rate than in the DGM, indicating that the gap-graded mixture could sustain loading for a longer duration during softening. The mechanism responsible for this behavior was considered to be the lack of intermediate sizes of aggregate in the GGM to interfere with the large ones during particle movement in dilation [48]. Further discussion in this respect is provided below by leveraging the simulation work.

**Figure 7:**
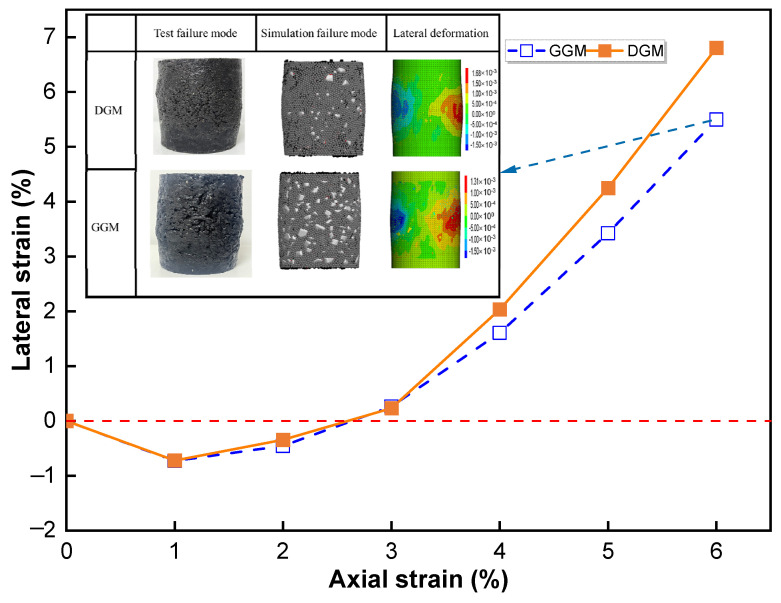
Volumetric deformation histories from the simulation (with the inset comparing the test and virtual specimens at the final strains).

Figure 7 presents the volumetric strain histories with respect to the axial deformation for the two mixtures from the simulation; the experimental data are not provided due to the unavailability of a measuring instrument for the radial deformation. In the simulation, the axial and radial strains were obtained for the middle third of the height of the virtual specimen, and specifically for the radial strain calculation, the corresponding shell elements’ displacements were lumped and averaged. Based on the volumetric deformation histories, both mixtures initially underwent densification, followed by volumetric expansion as they approached peak stress. At axial strains below 3%, the two mixtures exhibited quite similar volumetric behaviors. However, upon entering the dilation phase, the dense-graded mixture demonstrated a higher rate of volumetric expansion. In other words, the dense gradation rendered a greater volumetric sensitivity at the same axial deformations compared to the gap gradation. This observation aligns with previous experimental findings that the DGM softened more rapidly in the post-peak phase, as a greater dilation corresponds to more severe weakening of the aggregate structure.

### 4.2. Aggregate Movement Characteristics

The mechanical response of asphalt mixtures is a macroscopic manifestation of the internal framework formed by aggregates and asphalt mortar. For instance, the key contributions to the shear strength include aggregate interlocking, the internal cohesion of asphalt mortar, and the friction force between different components. When the contact stress is beyond the bond strength, contact failures occur, facilitating aggregate sliding and rotation. To characterize mesoscopic aggregate movement during dilation, the virtual specimen was evenly divided into six layers from bottom to top. Multiple measurement spheres were arranged in these layers to quantify the movement, and the data collected during loading were used in subsequent statistical analysis.

**Figure 8:**
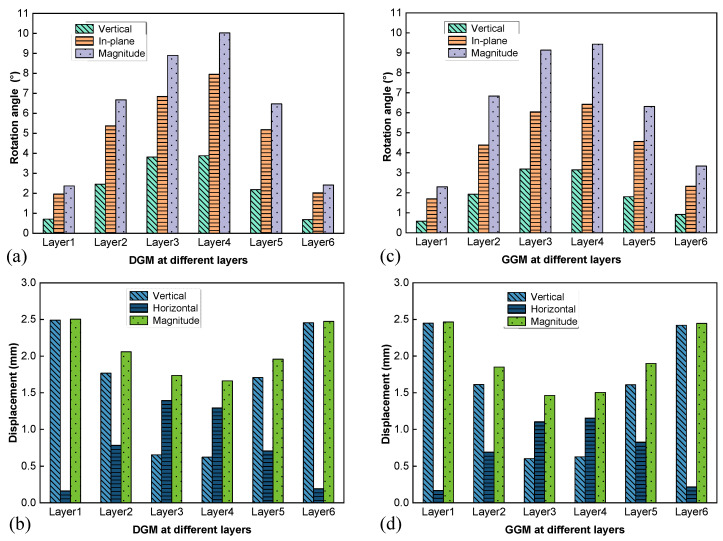
Aggregate movement at different layers: rotation angle (**a**) and displacement (**b**) for the DGM, and rotation angle (**c**) and displacement (**d**) for the GGM.

The aggregate movement characteristics in terms of rotation and displacement are presented in Figure 8. Note that the rotation angle was represented not only by the magnitude but also by its components in the horizontal plane (designated as in-plane) and in the vertical direction, as shown in Figure 8a,c, to facilitate examining the individual contributions to the lateral dilation. The displacement results were similarly decomposed for the same purpose, as shown in Figure 8b,d.

The two mixtures demonstrated similar profiles of rotation angle along the height (Figure 8a,c); the degree of rotation reached the peak at the middle height (layers 3 and 4) and decreased towards the two ends of the specimen. Moreover, for both cases, the primary contribution to the rotation was the in-plane component (with the rotation axis in the horizontal plane), which is closely related to the aggregates rolling radially over each other, hence the lateral expansion. As seen in Figure 8b,d, the two mixtures also yielded similar profiles of displacement, which differed from those of the rotation. Specifically, the displacement magnitudes reached the minima at the middle height and increased towards the two ends, which was expected since the virtual specimens were loaded symmetrically from the two ends. Interestingly, the vertical components followed the same trend as the magnitudes, while the horizontal components exhibited the opposite trend. Horizontal displacements peaked around the middle height, constituting the primary contributor to the overall particle translation. This dominant horizontal displacement served as the other important mechanism underlying the specimen bulging.

By comparing the two differently graded mixtures, it was noted that the DGM yielded more significant particle rotations and displacements in the horizontal plane at layers 3 and 4 (the middle height), which agreed with its higher volumetric expansion, as shown in Figure 7.

According to the particle packing theory [49], large particles that form the aggregate skeleton through interlocking are termed the main skeleton particles, whereas intermediate particles fill voids within the skeleton and provide structural stability. The fine particles (including mineral fillers) and asphalt binder, which comprise the asphalt mortar, are primarily responsible for cementation. A well-packed aggregate structure exhibits higher initial density and demonstrates superior performance in terms of rutting resistance and compaction [50]. In the literature, the concept of dominant aggregate size range (DASR) has been developed, referring to the range of sizes to form an interactive network of particles in continuous contact with each other [51]. For the two mixtures evaluated in this study, aggregate particles in the range of 4.75–9.5 mm accounted for the majority in each gradation and were considered as the main skeleton particles, while those in the range of 2.36–4.75 mm were treated as the interfering particles [49].

The porosity formed by the main skeleton particles is the key parameter that determines whether or not a particular gradation will result in a stable aggregate structure, and the interfering particles can greatly affect this structural stability. The volumes of these two particle grades in the virtual specimens were calculated to derive the disturbance factor:

(6) $$D_{F}=\frac{V_{IC}}{V_{DASR}}$$

where *D_F_* is the disturbance factor, *V_IC_* is the volume of the interfering particles, and *V_DASR_* is the volume of the main skeleton particles. The larger the disturbance factor, the more unstable the aggregate structure. The disturbance factor obtained for the DGM was 0.29, much higher than the 0.09 for the GGM. The dense gradation exhibited a greater disturbance effect, indicating a less stable aggregate structure in resisting loading at high temperatures, which explained the higher decreasing rate in the post-peak softening phase, as shown in Figure 6.

**Figure 9:**
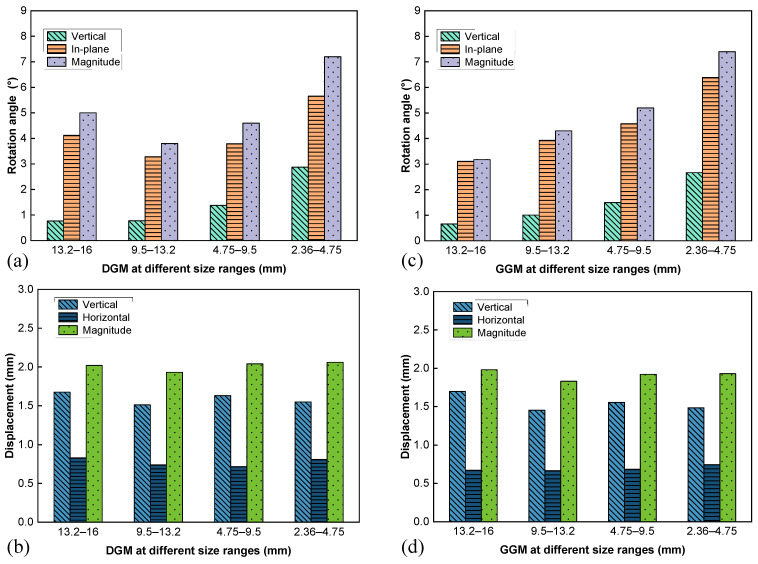
Aggregate movement at different size ranges: rotation angle (**a**) and displacement (**b**) for the DGM, and rotation angle (**c**) and displacement (**d**) for the GGM.

To further examine the influence of each aggregate grade on the skeleton structure, the movement characteristics of each size range were analyzed in terms of rotation and displacement, as illustrated in Figure 9. Similar to the previous layered analysis in Figure 8a,c, for both gradations the major component of the particle rotation was in the horizontal plane regardless of the size range, as noted in Figure 9a,c. Furthermore, smaller particles generally exhibited larger rotation angles due to there being fewer contacts and less restraints imposed by the surrounding aggregates. The highest rotation angles observed in the 2.36–4.75 mm size range for both mixtures confirmed the interfering nature of these particles, consistent with particle packing theory.

The distributions of displacement and its components were quite similar across the different grades for both mixtures, as seen in Figure 9b,d. The aggregate movement characteristics with respect to the size range did not exhibit patterns as clear and consistent as those with respect to the layer or specimen height. This observation was expected, as layered analysis is more effective in highlighting specimen bulging, while size analysis fails to capture overall bulging due to the smearing effect contributed by the particles with limited lateral expansion at the ends.

### 4.3. Crack Analysis

#### 4.3.1. Crack Development

**Figure 10:**
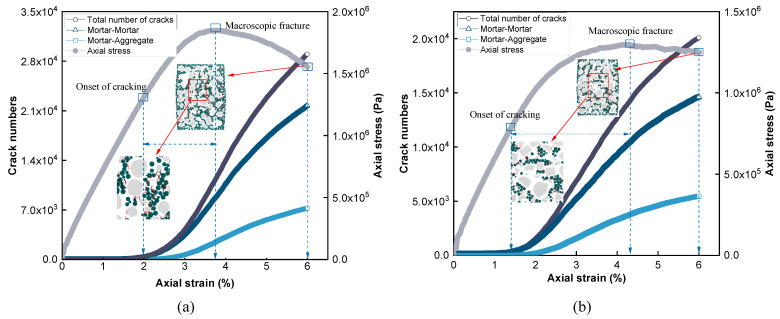
Crack development in the virtual specimens: (**a**) DGM and (**b**) GGM.

Upon reaching peak stress, contact failures likely occurred within the specimen, as evidenced by the crack initiation and propagation, as well as the loss of internal cohesion. In the numerical simulation, the internal damage of the virtual specimen was explored quantitatively by means of crack fragments developed at contacts, which have been shown to reasonably capture the contact failure mechanism in the dilation behavior [52]. Figure 10 shows the evolution of the number of cracks (failed contacts) during the deformation; the cracks were further categorized into contributions stemming from the mortar–mortar and mortar–aggregate contacts. For both gradations, failed contacts primarily originated in the mortar phase, indicating that shear failure is predominantly driven by a loss of internal cohesion. Compared to the GGM, the DGM generated more cracks in the mortar phase at the same axial deformation, particularly in the post-peak region, indicating a higher propensity for cohesion loss, consistent with the rapid stress drop after peak stress. In addition, the dense gradation also yielded a slightly higher number of cracks at the mortar–aggregate interface.

Figure 10 also includes vertical cross-sections of the virtual specimens at the final axial strain of 6%. These snapshots demonstrate that fewer cracks were generated in the mortar phase of the GGM. The range between the onset of cracking and the peak stress can be regarded as the process of damage accumulation until the eventual macroscopic fracture occurs that destabilizes the internal structure of the mixtures [52], as shown in Figure 10. Compared to the DGM, the gap-graded mixture provided a much wider deformation range for sustaining the damage accumulation before structural destabilization and softening, which was considered an advantage gained from the lower rate of cohesion loss.

The coordination number is an index used to reflect the topological relationship and the degree of compactness of particle stacking [53]. It has been pointed out that the strength of an aggregate skeleton is significantly affected by its coordination number [54]. This parameter can be calculated as:

(7) $$CN=\frac{\left(2N_{C}-N_{1}\right)}{\left(N_{P}-N_{0}-N_{1}\right)}$$

where *CN* denotes the coordination number, *N_C_* is the total number of contacts, *N*_1_ is the number of particles with one contact, *N*_0_ is the number of particles without a contact, and *N_P_* is the total number of particles.

**Figure 11:**
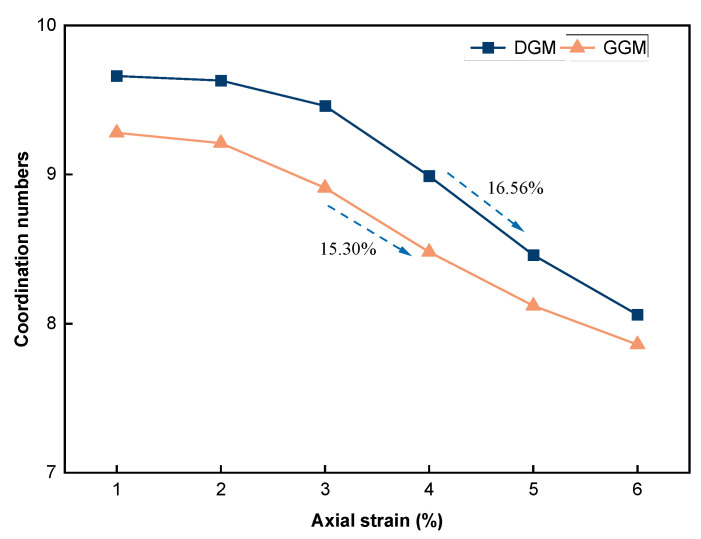
Coordination numbers along the deformation.

Figure 11 shows the evolution of coordination numbers for the two mixtures. The coordination number varied steadily in both cases in the initial stage of loading, suggesting a well-equilibrated arrangement of particles in the virtual specimens to start with. This observation confirms the effectiveness of the graduated expansion method adopted in model generation. The DGM exhibited reasonably higher coordination numbers along the deformation than the GGM as a result of the continuous gradation creating more interparticle contacts. For both mixtures, the decreasing trend of *CN* was considered a reflection of the increasing contact failures and dilation with deformation. Near and beyond the peak stresses at approximately 4% axial strain, *CN* decreased at the highest rate for both mixtures, as shown in Figure 11. Additionally, the dense gradation showed a slightly faster decrease in *CN*, which is consistent with the higher rates of crack development and cohesion loss in the DGM, as previously observed in Figure 10.

#### 4.3.2. Crack Distribution

**Figure 12:**
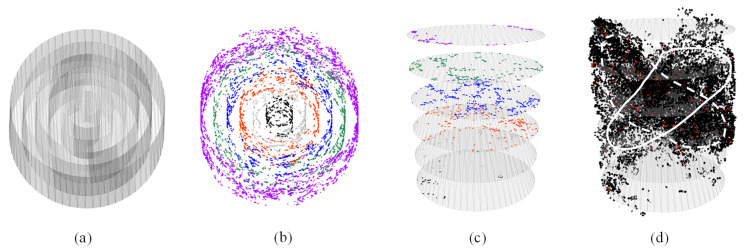
Distribution of crack fragments: (**a**) the cylindrical intersecting surfaces, (**b**) the radial distribution of cracks captured by the cylindrical surfaces as indicated by different colors, (**c**) the disk-shaped intersecting planes with the axial distribution of cracks, and (**d**) the overall distribution of cracks with the shear planes outlined by the white solid and dashed curves.

Contact failure between particles leads to the generation of cracks, and the spatial (radial and axial) distributions of crack fragments are of interest to investigate. For this purpose, different surfaces were generated to intersect the specimens and capture the failed contacts. For the radial distribution, a total of six cylindrical surfaces were created, denoted as Cyl1, Cyl2, …, and Cyl6 from the inside out. They all shared the same central axis and height with the virtual specimen but had increasing radii from 5 to 30 mm, with a 5 mm interval. This arrangement divided the specimen into coaxial cylindrical shells, as illustrated in Figure 12a,b. For the axial distribution, six disk surfaces were generated at the middle height of the individual layers, as used in the previous analysis, and were denoted as Disc1, Disc2, …, and Disc6 from the bottom to the top, as shown in Figure 12c,d. A visual inspection of Figure 12b,c indicates that the outmost shell and the middle two disks captured the most crack fragments, which is in line with the triaxial test in that the materials at the middle height towards the perimeter were more subjected to the effects of dilation (aggregate movement) and contact failures. Figure 12d depicts the overall distribution of cracks in the specimen with the X-shaped shear planes outlined, which is consistent with the general morphology of shear failure in triaxial testing.

**Figure 13:**
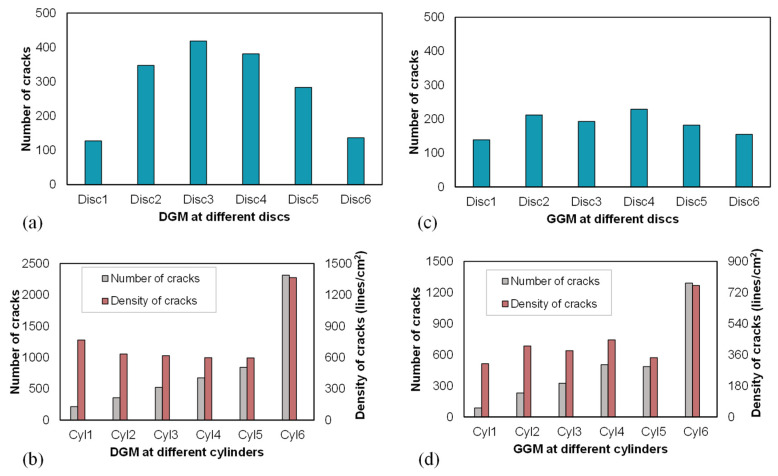
Distribution of cracks captured by the intersecting surfaces in the axial (**a**) and radial (**b**) directions for the DGM, and in the axial (**c**) and radial (**d**) directions for the GGM.

For the purpose of quantitative inspection, Figure 13 presents the distribution of the number of cracks captured by the intersecting surfaces in the radial and axial directions for the two mixtures at the end of the simulations. Compared to the GGM, the dense-graded mixture exhibited significantly more cracks around mid-height, as shown in Figure 13a,c. The more significant contact failures in the DGM were considered an important mechanism driving the higher degree of volumetric expansion, as noted earlier in Figure 7. The number of cracks intersected by the disk planes only slightly increased towards the middle height in the GGM, indicating a more stable aggregate structure provided by the gap gradation, consistent with the previous aggregate movement analysis, as shown in Figure 8.

Figure 13b,d show a progressive increase in the total number of cracks from Cyl1 to Cyl6 (from inside out) in both mixtures, as expected based on Figure 12b. Noting that an important contribution to this trend is the increasing surface area from inside out, the number density of the cracks was therefore calculated for a fair comparison. For both mixtures, the outermost cylindrical surface exhibited the highest crack number density, corresponding to the bulging behavior, as the peripheral materials experienced fewer constraints from the surroundings. The Cyl1 surface recorded the second-highest crack density at the specimen center for the DGM but the lowest for the GGM. Additionally, a side-by-side comparison revealed that the dense gradation consistently produced more cracks in the radial direction than the gap gradation, explaining the greater loss of internal cohesion and rapid post-peak softening in the DGM, as discussed earlier.

### 4.4. Contact Analysis

#### 4.4.1. Tensile and Compressive Contacts

**Figure 14:**
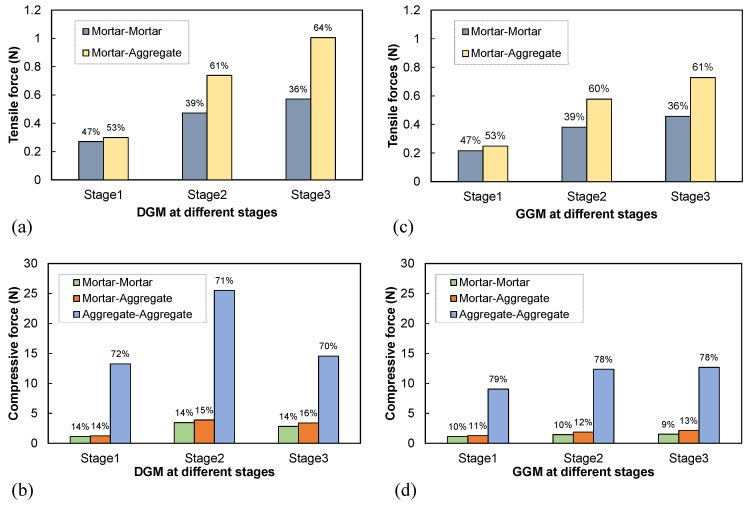
Contact forces between different components: tensile (**a**) and compressive (**b**) forces within the DGM, and tensile (**c**) and compressive (**d**) forces within the GGM.

The contact force between particles is an intrinsic manifestation of the mesoscopic load transfer path. To facilitate observing the evolution of contact forces along the dilation, the deformation history was divided into three stages according to the axial strains at 2% and 4%. Figure 14 shows the average tensile and compressive forces for different types of contacts and their percentages in each case. Tensile forces occurred only at contacts involving mortar and were significantly smaller in magnitude than compressive forces. As shown in Figure 14a,c, tensile forces at mortar–mortar and mortar–aggregate contacts increased with deformation in both mixtures, as more contacts experienced tension due to aggregate rearrangement under continuous loading. The forces at both types of contacts in the DGM were consistently greater than in the GGM. Further, for both mixtures, the mortar–aggregate interface accounted for a larger proportion than within the mortar phase in bearing the tensile forces. The percentage of tensile forces at the interface increased throughout deformation, particularly in the dense-graded mixture, consistent with the earlier observation of more cracks at the mortar–aggregate interface in the DGM, as shown in Figure 10.

The inter-aggregate contacts were the primary bearer of compressive forces and thus played a dominant role in the load transfer across the aggregate skeleton [16]. Compressive contact forces exhibited distinct evolution patterns between the two gradations, as shown in Figure 14b,d. In the DGM, compressive forces for all contact types peaked at stage 2 and declined in stage 3, whereas no significant changes occurred between these stages in the gap-graded mixture. The rapid reduction in compressive forces in the DGM can be attributed to its earlier entry into the post-peak softening phase at a lower strain level compared to the GGM, as well as its faster softening rate, as observed in Figure 10. The abrupt change in aggregate–aggregate contact forces, as shown in Figure 14b, may imply a pronounced weakening of the aggregate structure that corresponded to the macroscopic dilation. On the contrary, as seen in Figure 14d, the compressive contact forces in the GGM were more stably evolving during the latter two stages. Thus, the gap gradation provided greater structural stability despite its lower strength, as inferred from the compressive contact forces in Figure 14b,d. Notably, aggregate–aggregate contacts accounted for higher proportions in the GGM than in the DGM across all stages, suggesting that interparticle contacts and interlocking played a more significant role in the strength and stability of gap-graded mixtures.

#### 4.4.2. Anisotropy of Contact Forces

**Figure 15:**
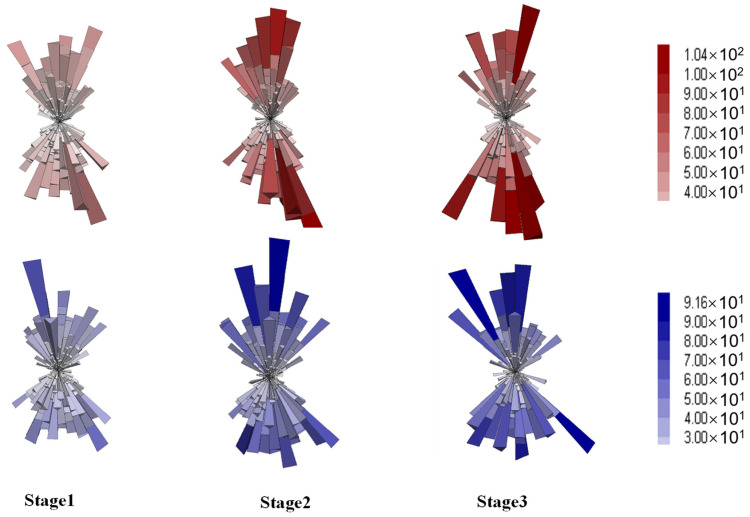
Distribution of the normal contact forces (red for DGM and blue for GGM).

**Figure 16:**
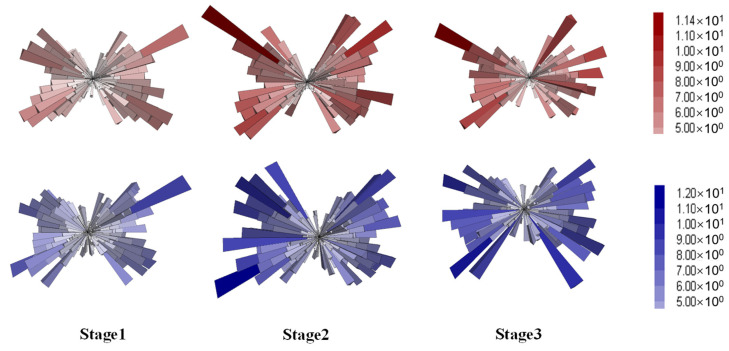
Distribution of the shear contact forces (red for DGM and blue for GGM).

Asphalt mixture is a highly heterogeneous composite material composed of irregularly shaped and sized aggregate particles and asphalt binder, which exhibit sharp contrasts in mechanical properties, such as stiffness and temperature sensitivity. To investigate the effect of the aggregate skeleton on the anisotropy of load transfer and force chains from a mesoscopic perspective, three-dimensional distribution diagrams and deviator fabric of contacts were employed. Contact forces between particles can be categorized into normal and shear components, with their distribution diagrams at different stages shown in Figure 15 and Figure 16, respectively.

Each elongated column represents the average contact force in a specific direction, with the size and color indicating the force magnitude. For both mixtures, the normal contact forces demonstrated increasing magnitudes and directionality with the axial deformation, as shown in Figure 15. Compared to the gap gradation, the DGM exhibited higher force magnitudes, in agreement with the higher shear strength and larger coordination numbers provided by the dense gradation. Compared to normal forces, the shear contact forces showed similar spatial distributions and magnitudes for both mixture types, as illustrated in Figure 16. Additionally, the diagrams highlighted the X-shaped weak zones that are prone to shear cracking, consistent with the shear planes observed earlier in Figure 12d.

**Figure 17:**
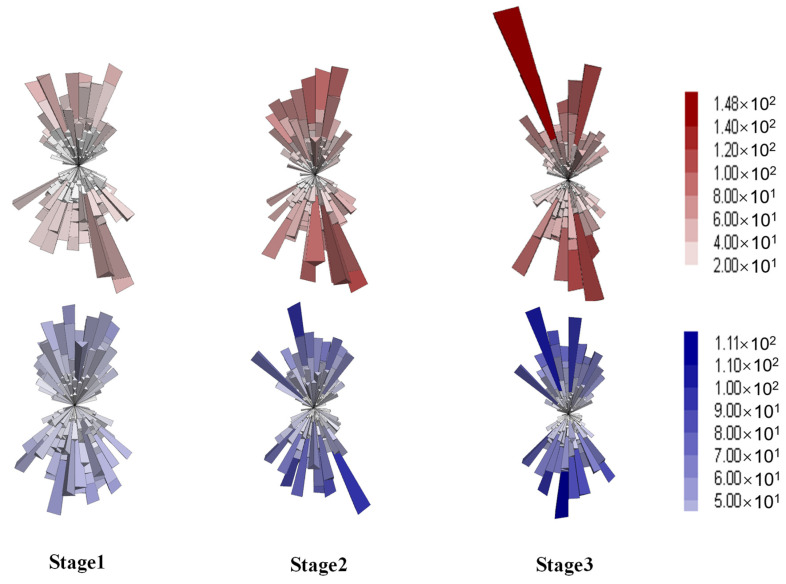
Distribution of the strong contact forces (red for DGM and blue for GGM).

**Figure 18:**
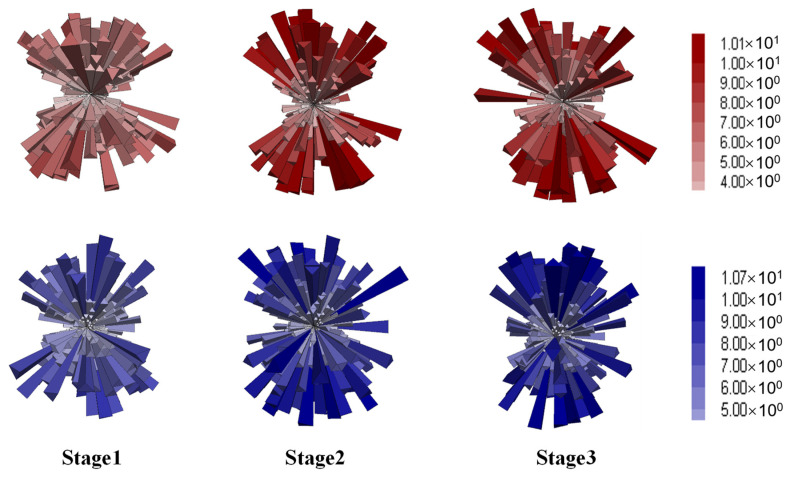
Distribution of the weak contact forces (red for DGM and blue for GGM).

The contact forces were further analyzed by categorizing them into strong (above the average value) and weak (below the average value). Considering that the normal contacts were more discriminative between the two gradations, according to Figure 15, this analysis was performed on the normal forces. The resulting distributions for the strong and weak contacts are provided in Figure 17 and Figure 18, respectively.

The strong contacts, as seen in Figure 17, exhibited overall distributions similar to those of the total normal forces, evidencing the primary role in bearing the external loading [53]. As deformation increased, the directionality of strong forces became more aligned with the loading direction. Conversely, the weak forces were distributed in a more spherical pattern, as shown in Figure 18, corresponding to volumetric deformation. In summary, the distribution analysis indicated that strong forces played a dominant role in bearing external loads and resisting axial compression, while weak contacts were primarily associated with volumetric expansion [55].

A quantitative approach towards the distributions of contact forces can be achieved via the deviator fabric of the interparticle contacts, determined by [35,56]:

(8) $$F_{ij}=\frac{1}{N_{c}}\sum_{k=1}^{N_{c}}n_{i}^{k}n_{j}^{k}$$

(9) $$D_{f}=\frac{1}{\sqrt{2}}\sqrt{{\left(\lambda_{1}-\lambda_{2}\right)}^{2}+{\left(\lambda_{1}-\lambda_{3}\right)}^{2}+{\left(\lambda_{2}-\lambda_{3}\right)}^{2}}$$

where *N_c_* is the total number of interparticle contacts in the assembly; *n^k^* is the unit normal contact vector of the *k*-th particle with *i* and *j* = 1, 2, and 3; and *F_ij_* is referred to as the fabric tensor of the contact network and has three eigenvalues, designated as *λ*_1_, *λ*_2_, and *λ*_3_, that are used to calculate the deviator fabric *D_f_*. The larger the deviator fabric, the greater the degree of contact anisotropy [33,54].

**Figure 19:**
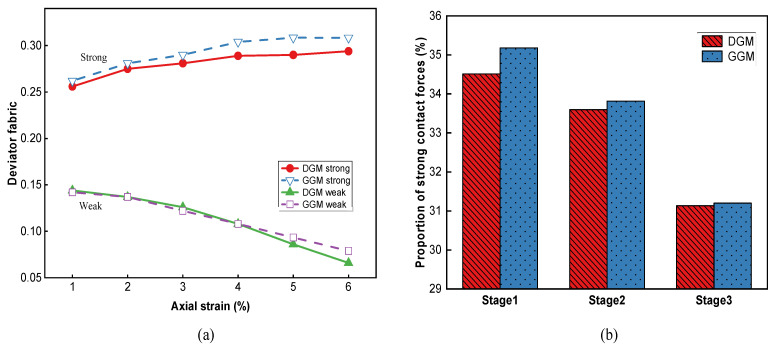
Anisotropy analysis of contacts: (**a**) deviator fabric for the strong and weak contacts, and (**b**) the percentages of strong contacts at different stages.

Figure 19a shows the evolution of the *D_f_*-values for the two mixtures, which exhibited opposite trends between the strong and weak contacts. The deviator fabric increased steadily with axial deformation, as evident from the more aligned spatial distributions in Figure 17, and stabilized beyond 4% strain during the softening phase. The *D_f_*-values of the GGM were slightly higher than those of the dense gradation throughout the deformation, which highlighted the more important load-bearing role of aggregate skeleton in the gap-graded mixture (especially in the post-peak softening phase). Compared to strong contacts, weak contact forces produced significantly smaller *D_f_* -values that continued to decrease with deformation. This decreasing trend of *D_f_* (that is, increasing isotropy) is believed to be a mesoscopic outcome of the volumetric expansion. Between the two gradations, the *D_f_*-values were quite similar in the first two stages, but the DGM started to deviate downwards in the last softening stage.

Figure 19b presents the percentages of strong contacts within the virtual specimens throughout the three stages. For both mixtures, the proportion of strong contacts decreased throughout the whole deformation history as a result of continuous contact failures and particle rearrangement. The GGM exhibited higher proportions of strong contacts at all stages than the DGM, indicating that the aggregate skeleton played a more important role in the strength and stability of the gap-graded mixture, consistent with previous observations.

## 5. Conclusions

This study presented a mesoscopic investigation into asphalt mixtures with the aim of understanding the shear dilation behavior underlying high-temperature performance against permanent deformation. The triaxial strength test was simulated by coupling the discrete and finite element methods considering realistic 3D aggregate shapes and gradations. For allowing contact failures among the mortar units and at the mortar–aggregate interfaces, the linear parallel bond model was mixed with the Burgers or linear model through random distribution in the virtual specimens. The flexible boundary, as prescribed typically by a latex membrane in laboratory testing, was treated by finite shell elements in the simulation. Model validation was performed by comparing the resulting stress–strain data against those from the laboratory testing. In order to assess and highlight the role of aggregate gradation in resisting shear dilation, both the dense and gap gradations with 13 mm NMAS were considered in the experiment and simulation. A systematic mesoscopic investigation was then performed from the perspectives of particle movement, crack development and distribution, and interparticle contacts. The following conclusions were drawn based on the findings:

- With the same asphalt binder, the gap-graded mixture yielded lower strengths in the experiment at all confining pressures considered, which was attributed to its higher binder content (as required by the gap gradation) not being compensated for by enhanced viscosity.
- Compared to the dense-graded mixture, GGM entered the post-peak softening phase at a larger strain level; it softened at a much lower rate and exhibited lower volumetric expansion, suggesting a higher structural stability at high temperatures.
- The specimen bulging, which was most prominent around the middle height, was driven by the dominant in-plane or horizontal components of particle rotation and translation. The structural stability of the gap-graded mixture was attributed to its lower magnitudes in both components of particle movements and also to its inclusion of less interfering particles in the size range of 2.36–4.75 mm.
- Contact failure-induced cracks primarily occurred within the mortar phase. The accumulation of cracks and the reduction in coordination number due to contact failures were both faster in the DGM, suggesting a more rapid loss of internal cohesion in agreement with the quicker drop in the axial stress after the peak. The cracks were more concentrated at the middle height and towards the perimeter of the virtual specimens; lower numbers or densities of cracks were noted within the gap-graded mixture.
- In both mixtures, the mortar–aggregate interfaces were the major bearer of tensile contact forces, but the average forces and the accumulation of cracks at the interfaces were considerably higher in the DGM. The compressive contact forces were dominated by the inter-aggregate contacts, especially in the case of the GGM; the gap gradation yielded lower and yet more steadily varying compressive forces.
- Anisotropy analysis on the contact forces suggested that the distribution of strong forces was more pertinent to resisting the axial loading, while that of the weak forces was more relevant to the volumetric expansion. It was inferred from the contact anisotropy that the aggregate skeleton played a more significant role in the strength and stability of gap-graded mixtures.

By the combined experimental and simulation method, it was pointed out in this study that the gap gradation could produce higher structural stability at elevated temperatures for the asphalt mixture to resist severe permanent deformation. In addition, the aggregate stability as revealed in the post-peak softening phase was correlated with the distribution of cracks (due to contact failures) and contact forces. Future work may extend the present effort to additional material factors and repeated loading for enhanced simulations of the rutting performance of asphalt mixtures.

## Figures and Tables

**Table 1:** Asphalt binder properties.

Properties	Test Methods	Requirements	Measurements
Penetration (0.1 mm)	T0604/JTG E20-2011 [29]	60–80	67.8
Softening point (°C)	T0606/JTG E20-2011	≥46	47
Ductility (cm)	T0605/JTG E20-2011	≥100	150
Dynamic viscosity (Pa⋅s)	T0620/JTG E20-2011	≥180	224
Density (g/cm^3^)	T0603/JTG E20-2011	As measured	1.035

**Table 2:** Mineral aggregate properties.

Properties	Sieve Size Ranges (mm)			Requirements
	0~5	5~10	10~15	
Apparent density (g/cm^3^)	2.73	2.74	2.84	≥2.6
Crushing value (%)	/	/	11.1	≤26
Water absorption rate (%)	1.6	1.2	0.9	≤2
Flat and elongated particles (%)	/	14.6	12.8	≤18

**Table 3:** The number of balls required for each sieve size in the DGM.

Model Parameters	Sieve Size Ranges (mm)			
	2.36~4.75	4.75~9.5	9.5~13.2	13.2~16
Volume fraction (%)	8.28	28.12	15.56	3.93
Total volume (mm^3^)	21,091.64	71,549.81	39,607.85	10,008.47
Average diameter (mm)	3.56	7.13	11.35	14.60
Volume of each ball (mm^3^)	23.52	189.38	765.57	1629.51
Number of balls	896	378	51	6

**Table 4:** Mesoscopic parameters for the Burgers model.

Mesoscopic Parameters	Units	DGM	GGM
Kkn	Pa·m	4.93 × 10^3^	3.67 × 10^3^
Ckn	Pa·m·s	8.83 × 10^5^	6.58 × 10^5^
Kmn	Pa·m	2.60 × 10^4^	1.93 × 10^4^
Cmn	Pa·m·s	1.52 × 10^7^	1.13 × 10^7^
Kks	Pa·m	1.97 × 10^3^	1.47 × 10^3^
Cks	Pa·m·s	3.53 × 10^5^	2.63 × 10^5^
Kms	Pa·m	1.03 × 10^4^	7.73 × 10^3^
Cms	Pa·m·s	6.09 × 10^6^	4.54 × 10^6^

**Table 5:** Mesoscopic parameters for the LPB model.

Pb_coh (MPa)	Pb_ten (MPa)	Pb_fa (°)	Pb_emod (GPa)	Emod (GPa)	μ
0.53	1.43	40	0.0225	0.15	0.35

**Table 6:** Test results.

Mixture Types	Average Strengths at Different Pressures (kPa)			Mohr–Coulomb Parameters	
	0	138	276	*c* (kPa)	*j* (°)
DGM	1047	1696	2341	242.2	40.4
GGM	572	1442	1783	146.3	40.8

## Data Availability

The original contributions presented in this study are included in the article. Further inquiries can be directed to the corresponding author.
